# Detergent resistant membrane-associated IDE in brain tissue and cultured cells: Relevance to Aβ and insulin degradation

**DOI:** 10.1186/1750-1326-3-22

**Published:** 2008-12-31

**Authors:** Ayelén Bulloj, María C Leal, Ezequiel I Surace, Xue Zhang, Huaxi Xu, Maria D Ledesma, Eduardo M Castaño, Laura Morelli

**Affiliations:** 1 Fundación Instituto Leloir. IIBBA-CONICET. Ave. Patricias Argentinas 435, Ciudad de Buenos Aires C1405BWE, Argentina; 2 Burnham Institute for Medical Research, La Jolla, California 92037, USA; 3 Centro de Biología Molecular Severo Ochoa, 28049 Madrid, España

## Abstract

**Background:**

Insulin degrading enzyme (IDE) is implicated in the regulation of amyloid β (Aβ) steady-state levels in the brain, and its deficient expression and/or activity may be a risk factor in sporadic Alzheimer's disease (AD). Although IDE sub-cellular localization has been well studied, the compartments relevant to Aβ degradation remain to be determined.

**Results:**

Our results of live immunofluorescence, immuno gold electron-microscopy and gradient fractionation concurred to the demonstration that endogenous IDE from brain tissues and cell cultures is, in addition to its other localizations, a detergent-resistant membrane (DRM)-associated metallopeptidase. Our pulse chase experiments were in accordance with the existence of two pools of IDE: the cytosolic one with a longer half-life and the membrane-IDE with a faster turn-over. DRMs-associated IDE co-localized with Aβ and its distribution (DRMs vs. non-DRMs) and activity was sensitive to manipulation of lipid composition in vitro and in vivo. When IDE was mis-located from DRMs by treating cells with methyl-β-cyclodextrin (MβCD), endogenous Aβ accumulated in the extracellular space and exogenous Aβ proteolysis was impaired. We detected a reduced amount of IDE in DRMs of membranes isolated from mice brain with endogenous reduced levels of cholesterol (Chol) due to targeted deletion of one seladin-1 allele. We confirmed that a moderate shift of IDE from DRMs induced a substantial decrement on IDE-mediated insulin and Aβ degradation in vitro.

**Conclusion:**

Our results support the notion that optimal substrate degradation by IDE may require its association with organized-DRMs. Alternatively, DRMs but not other plasma membrane regions, may act as platforms where Aβ accumulates, due to its hydrophobic properties, reaching local concentration close to its Km for IDE facilitating its clearance. Structural integrity of DRMs may also be required to tightly retain insulin receptor and IDE for insulin proteolysis. The concept that mis-location of Aβ degrading proteases away from DRMs may impair the physiological turn-over of Aβ in vivo deserves further investigation in light of therapeutic strategies based on enhancing Aβ proteolysis in which DRM protease-targeting may need to be taken into account.

## Background

Insulin degrading enzyme (IDE) is a thiol zinc-metallopeptidase involved in the hydrolysis of several peptides including insulin and amyloid β (Aβ) [1,2] and implicated in regulating cellular growth and differentiation [3]. Subcellularly, IDE is primarily found in cytosol [4], peroxisomes [5] and plasma membrane (PM) [6]. In addition, a secreted form of IDE has been found in conditioned media of cell lines and primary neuronal cultures [7]. The primary structure of IDE does not indicate the presence of a signal peptide for entering the secretory pathway or any predicted membrane-associated domains that could explain its association with membranes and its secretion.

Aβ is the major component of amyloid plaques, a pathological hallmark of Alzheimer's disease (AD). In addition to IDE, Aβ can be degraded by several proteases, including neprilysin (NEP), endothelin converting enzyme-1 (ECE-1), plasmin, and matrix metalloproteinases (MMPs) [8]. There is compelling evidence supporting the physiological role of IDE in the degradation and homeostasis of Aβ and insulin. Recent studies in animal models support a pivotal role of IDE in Aβ catabolism. Firstly, diabetes-associated mutations in rat IDE impair Aβ degradation [9]. Secondly, cerebral Aβ levels are elevated and hyperinsulinemia and glucose intolerance are seen in IDE knockout mice [10]. Finally, overexpression of IDE in transgenic AD mouse models decreases Aβ levels and retards the formation of amyloid plaques [11].

Several reports indicate that insulin degradation by IDE requires binding to its receptor, reported to reside in DRMs [12] in all the tissues and cell types studied [13-15].

Noteworthy, the mechanisms by which IDE accesses each substrate for degradation are not clear and neither the functional significance nor a systematic study of the association of endogenous IDE with membranes in cultured cells or brain tissue have been explored so far. Therefore, here we asked whether there is a pool of proteolytically active IDE associated with plasma membrane DRMs and its participation in Aβ and insulin degradation.

## Methods

### Antibodies and chemicals

Anti-IDE polyclonal (BC2) and monoclonal (1C1 and 3A2) antibodies were generated and characterized in our laboratory as described [16,17]. All of them are reactive against human, rat and mouse IDE. BC2 recognized linear epitopes within amino acids 97–273 of rat IDE, while both monoclonal antibodies (isotype IgG1a) are reactive for different epitopes within residues 262–530. Rabbit polyclonal S40 antiserum was provided by Dr. Mikio Shoji (Okayama University, Okayama, Japan) it is specific for Aβ-40 and does not recognize APP. Rabbit anti-APP C-terminal (AB5352) was from Chemicon. Purified monoclonal antibody against flotillin-1 was purchased from BD Transduction Laboratories. Mouse monoclonal antibody anti-tubulin and polyclonal antibody anti-actin were from Sigma. When indicated, experiments were performed in the presence of a protease inhibitor cocktail defined as: 10 mM PMSF, 20 μM leupeptin, 20 μM aprotinin, 50 μM thiorphan and 50 μM phosphoramidon with or without metalloproteinase inhibitors (5 mM EDTA and 1 mM 1,10 phenanthroline).

### Cell cultures

N2a cells (mouse neuroblastoma) from the American Type Culture Collection (ATCC) # CCL-131 were cultured in 50% Dulbecco's modified Eagle's medium (DMEM) and 50% Opti-MEM (Invitrogen) supplemented with 5% fetal bovine serum (FBS). N2aSW cells (N2a cells stably expressing human APP Swedish mutation [18] and N2aEGFP (N2a cells stably expressing the vector pEGFP), were cultured in the above N2a culture media supplemented with 400 μg/ml of G418 (Sigma). Primary cultures of hippocampal neurons were prepared from Wistar rat embryos of 17–18 days. Briefly, cells from hippocampus were dispersed with trypsin (0.24 mg/ml, GIBCO) followed by addition of Advanced-DMEM (GIBCO) supplemented with 5% FBS. Cells were washed twice with Hanks Balanced Salt solution (HBSS, GIBCO) and plated in Neurobasal medium (NB, GIBCO) containing 2% B27 supplement (GIBCO) and 0.5% glutamine (GIBCO).

### Tissues

Frozen postmortem brain samples from a previously characterized FAD patient [19] were obtained from the Department of Pathology, Hospital Santojanni, under the approval of the Institutional Ethics Committee. Adult intact female Wistar rats (200–250 g) were maintained on a 12 hour light/dark cycle, fed with standard diet, and housed according to the National Institutes of Health Guide for the Care and Use of Laboratory Animals. Animals were killed by decapitation at random points of the estrous cycle and the brain processed as described below. Heterozygous breeding pair mice with targeted deletion of one seladin-1 allele were kindly provided by Dr. E. Feinstein (Quark Pharmaceuticals Inc.). These mice were generated for Quark Pharmaceuticals by Lexicon Corp. under the service agreement. Seladin-1 deficient mice were bred and genotyped as previously described [20]. All animal experiments and husbandry were performed in compliance with national guidelines. The brains of Seladin-1 heterozygous (+/-) and wild type (+/+) mice were analyzed at 3 weeks of age.

### Immunoelectron Microscopy of endogenous IDE

Confluent cultures were washed and fixed with 2% p-formaldehyde (PFA)/0.5% glutaraldehyde. Cells were recovered with a cell-scraper and extensively washed with PBS. Pellets were dehydrated in ethanol and propylene oxide, embedded in LR White (Polysciences, Inc. Warrington, PA USA) and polymerized. After thin sectioning (750–900 Ǻ) samples were collected on carbon-formvar-coated nickel grids and processed using the immunogold technique. Briefly, grids were sequentially blocked with 0.02 M glycine and 1% bovine serum albumin (BSA)/5% normal goat serum in PBS and labeled with primary antibodies for 45 minutes at room temperature. After washing grids were incubated with anti-mouse and/or anti-rabbit IgG conjugated to colloidal gold particles of 15 nm and 6 nm, respectively (Electron Microscopy Sciences, Hatfield, PA USA). Control labeling was performed with a non-related mouse IgG instead of the primary antibody. Sections were post-fixed with 2% PFA/0.5% glutaraldehyde, washed, stained with 2% uranyl acetate and observed in a JEOL 1200 EX electron microscope (Akishima, Tokyo, Japan).

### DRMs and IDE live staining immunofluorescence

DRMs were stained on living N2a cells and primary hippocampal neurons with Vybrant Lipid Raft Labeling Kit (Molecular Probes-Invitrogen) following the manufacturer's instructions. Alexa fluor 594 conjugated to cholera toxin sub-unit B (CT-B) was used to label gangliosides on the PM followed by incubation with anti-CT-B polyclonal antibody in order to "patch" DRMs. For staining endogenous IDE associated to PM, cells were incubated with 0.5 mg/ml of 1C1 and 3A2 anti-IDE monoclonal antibodies for 1 hour at 4°C, extensively washed, and probed with anti-mouse Alexa 488 (green) or anti-mouse Cy3 (red) antibody for 1 hour at 4°C. Cells were then fixed with 4% PFA and mount on fluorescent mounting medium (DakoCytomation). Double-labeling analysis was performed by laser scanning confocal microscopy imaging of slides on a LSM5 Pascal Zeiss (Oberkochen) microscope using a three-frame filter and a Zeiss LSM5 image examiner. Assessment of co-localization of CT-B and IDE immunoreactivity was performed using the Image-Pro Plus software (Media Cybernetics)

### Subcellular fractionation

Cells were re-suspended in hypotonic buffer (10 mM KCl; 10 mM Tris-HCl; 1.5 mM MgCl_2_; 1 mM PMSF; pH 7.5) passed through a 21G-gauge needle several times and centrifuged at 4,000 × g for 5 minutes to pellet nuclei and nuclei-associated structures, including Golgi and endoplasmic reticulum membranes. The supernatant was centrifuged at 100,000 × g for 60 minutes in a 50 Ti rotor (Beckman) at 4°C to obtain a pellet containing the PM-enriched fraction as previously described [21]. Pellets were exhaustively washed in 0.5 M Na_2_CO_3_, pH 11 and centrifuged at 100,000 × g at 4°C for 1 hour. Both, PM-enriched and cytosolic fractions were solubilized in NP-40 lysis buffer (50 mM Tris-HCl pH 7.5; 150 mM NaCl; 0.5% NP-40) and stored at -80°C for further analysis.

### Determination of cytosolic and membrane-associated IDE turnover

Cells were labeled in methionine-depleted medium (Invitrogen) containing [^35^S]methionine (250 μCi/mL, GE Biosciences). For pulse-chase experiments, after labeling for 30 minutes cells were rinsed in PBS and incubated in culture medium for the chase after: 48, 72 and 96 hours for cytosolic IDE and 0.5; 1; 3; 6, 7.5; 11; 24 and 48 hours for membrane associated IDE, respectively. After each time-point, conditioned medium was recovered and cells were rinsed in PBS, scraped and processed as described above for sub-cellular fractionation except that PM-enriched pellets were resuspended in PBS/0.5% Triton X-100. Immunoprecipitations of IDE from cytosol and PM-enriched fraction were performed by incubation with 30 μL of Protein G-Sepharose (GE Bioscience) and 1C1/3A2 (specific anti-IDE monoclonal antibodies). Pellets were collected with a brief centrifugation, washed in 50 mM Tris-HCl pH 7.5; 120 mM NaCl; 1% NP-40; 0.1% SDS and analyzed by Tris- N-[2-hydroxy-1,1-bis (hydroxymethyl)ethyl-]glycine (Tricine) polyacrylamide gel electrophoresis (SDS-PAGE) on a 7.5% gel, and subjected to fluorography. The amount of radiolabeled IDE was estimated with a Storm 840 PhosphorImager (GE Bioscience). Each value of remaining radiolabeled IDE was referred to the maximum value obtained during the chase. The first-order decay constant (k) was calculated from one phase exponential decay curves using the form y = e(-kt) generated with the program GraphPad Prism 4. The half-life of IDE was determined according to the formula t1/2 = 0.693/k.

### Manipulation of lipid composition from PM-enriched fractions

Cells grown to confluence were washed once with PBS and incubated with 5 mM methyl-β-cyclodextrin (MβCD) (Sigma) in serum-free medium for 30 minutes at 37°C. Chol replenishment of MβCD-treated cells was performed by incubation with 1 mM water soluble Chol (Sigma) for 30 minutes at 37°C. The total lipid extracts were analyzed by thin-layer chromatography (TLC) followed by chemical detection [22]. Identification of lipids separated by TLC was accomplished by co-migration with standard lipids. The relative amounts of each lipid were determined by densitometry using the computer software Gel-Pro Analyzer 3.1 (Media Cybernetics). Cholesterol level in total cellular membrane was measure using a standar protocol previously described [23].

### Quantification of extracellular endogenous Aβ

N2aSW cells were treated or not with 5 mM MβCD in the presence of a protease inhibitors cocktail and supernatants were collected after 12 and 24 hours. To assess if APP processing was affected by MβCD treatment, endogenous levels of APP C-terminal fragments were determined in the cellular pellets by western blot as described below. Aβ40 levels were quantified in the supernatants using a commercially available ELISA kit (Covance Research Products) following the manufacturer's instructions.

### Cell surface degradation assay of extracellular Aβ

Synthetic Aβ 1–40 was obtained from Bachem (Torrance) and iodinated by the Chloramine-T method as described [24]. For cell surface degradation assay, 30,000 cpm of [^125^I]-Aβ40 (specific activity 15 μCi/μg) were added per milliliter of medium to 90% confluent control and MβCD-treated cells plated in 100 mm dishes and incubated at 37°C for 15 minutes in the presence of protease inhibitors as described above. Supernatants were removed and treated with 15% trichloroacetic acid to precipitate undegraded [^125^I]-Aβ40. The precipitated samples were centrifuged, and the amount of cpm in the pellet (intact peptide) was counted. Degradation was expressed as percentage compared to control referred as [^125^I]-Aβ40 incubated in cell culture medium free of cells.

### Isolation of DRMs and DSMs fractions from N2a cells, rat and human brain

Nearly confluent N2a cells grown in 100 mm dishes were washed in PBS/9% sucrose/protease inhibitor cocktail. Cellular homogenates were obtained after 10 strokes in a dounce homogenizer and 10 passages though a 22 G gauge needle syringe on ice. Samples were centrifuged for 10 minutes at 4°C and 700 × g and the supernatants further centrifuged at 100,000 × g for 1 hour at 4°C to isolate the membrane fraction. The final pellet was resuspended in MES buffer saline (MBS) containing 1% Triton X-100, passed through a 27 G × 1/2 -gauge needle 10 times and incubated 30 minutes at 4°C. For sucrose density gradient centrifugation, membrane lysates were adjusted to 60% (w/v) final concentration of sucrose (final volume 2 ml) and overlaid with 5 ml 35% and 5 ml 5% (w/v) sucrose in MBS containing 0.5% Triton X-100. Samples were centrifuged in a SW 41-Ti rotor (Beckman) at 100,000 × g at 4°C for 20 hours. Rat and human brain tissues were processed as described [25]. Briefly, membranes were isolated after sonication in MBS/protease inhibitor cocktail and centrifugation at 100,000 × g at 4°C for 90 minutes. The final pellet was resuspended in MBS containing 2% Triton X-100 and incubated for 2 hours at 4°C. The lysate was then loaded onto a sucrose gradient and processed as described above for cells. Fractions were collected from the top of each tube. For gradient characterization, protein level was measured using a bicinchoninic acid assay (Pierce). In each fraction, the density was calculated by the refractive index and the alkaline phosphatase activity by the generation of p-nitrophenol from nitrophenylphosphate monitored at 405 nm. DRMs (fractions 3 and 4) were defined by 3 criteria: low-density, positive alkaline phosphatase activity and the presence of flotillin-1 immunoreactivity. By contrast, DSMs corresponded to high density fractions (8 and 9) lacking alkaline phosphatase activity and flotillin-1 immunoreactivity.

### Quantification of endogenous Aβ in DRMs and DSMs from human brain

DRM and DSM fractions were mixed with 150 mM sodium citrate (pH 7.0) at a 8:1 (vol:vol) ratio and centrifuged at 100,000 × g for 1 hour in a SW-40 Ti rotor (Beckman) at 4°C to pellet membranes and exchange the buffer. The supernatant was discarded and the pellet was sonicated in reaction buffer containing 150 mM sodium citrate, pH 7.0/2% Brij-35, and complete protein inhibitor PI (Sigma). Aβ42 levels were determined using a commercially available ELISA kit (Covance Research Products) following the manufacturer's instructions.

### Immunoprecipitation-based IDE activity assay

IDE was immunoprecipitated from DRMs and DSMs fractions with 1C1 and 3A2 (anti-IDE monoclonal antibodies). Monoclonal anti-actin was used as a negative control. Immunocomplexes were isolated with protein G-Sepharose (GE Biosciences) and then washed with ice-cold buffer containing the protease inhibitor cocktail. Beads were then incubated for 4 hours in 50 μl degradation buffer (0.1 M phosphate, pH 7) containing 65,000 cpm of [^125^I]-insulin (specific activity, 300 μCi/μg; kindly provided by Edgardo Poskus, University of Buenos Aires) or [^125^I]-Aβ (specific activity, 20 μCi/μg) as described [16], in the presence of the protease inhibitor cocktail with or without metalloproteinase inhibitors, respectively. After incubation, beads were centrifuged at 1,500 × g for 5 minutes and 20 μl of the supernatant were removed, mixed with an equal volume of 8% SDS Laemmli sample buffer, boiled, and resolved by 15% Tris-Tricine SDS-PAGE under non-reducing conditions. The amount of intact remaining [^125^I]-insulin or [^125^I]-Aβ, as compared to the undegraded radiolabeled peptide in the negative control sample, was estimated with a Storm 840 PhosphorImager (GE Bioscience) and values expressed as percentage of intact peptide.

### Western Blotting

Proteins were resolved using 10% Tris-Tricine gels transferred to polyvinylidenedifluoride (PVDF) membranes (GE Bioscience) and incubated with primary antibodies (BC2, anti-tubulin, anti-flotillin-1 or anti-C-terminal APP, respectively). Immunoreactivity was detected with anti-rabbit or anti-mouse horseradish peroxidase-labeled IgG (Dako) and enhanced chemiluminescence ECL Plus (GE Bioscience). Immunoblots were scanned with Storm 840 and analyzed with ImageQuant 5.1 software (GE Bioscience).

### Statistical Analysis

All values are presented as means ± SE. ANOVA was used to determine differences among groups. Where a significant difference was indicated, the Tukey test was used to determine significant differences between groups. p < 0.05 was considered to be statistically significant.

## Results

### IDE resides in DRMs of the plasma membrane

**Figure 1:**
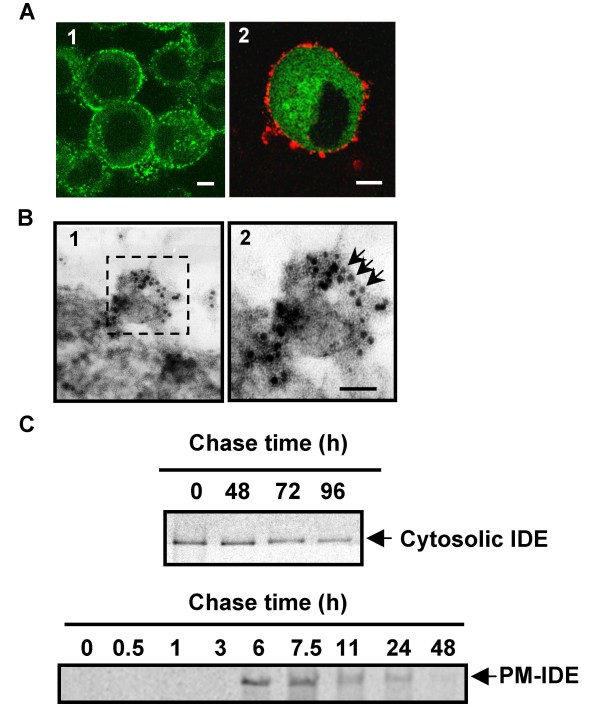
**Plasma membrane and cytosolic IDE have different turn-over**. **A**-Immunofluorescence of PM-associated endogenous IDE in living N2a wild type (N2WT) (panel 1) and Na2WT-EGFP (panel 2) cells using 1C1 monoclonal antibody and Alexa 488 (green) or Cy3 (red) anti-mouse IgG, respectively. Expression of EGFP was visualized based on the EGFP fluorescence (green) and labeled the cytosolic compartment. Scale bars: 5 μm. **B**-Immunogold electron microscopy on cryosections of N2aSW cells showed a cluster of IDE molecules at the PM (panel 1) which were more evident at higher magnification of the framed region (panel 2). Scale bar, 50 nm. **C**- Representative phosphorimaging of the remaining IDE during the chase period in cytosol (upper panel) and PM (lower panel). N2aWT cells were pulse-labeled with [^35^S]-methionine for 30 minutes and chased for the indicated times (0 to 96 hours). Cytosolic and PM fractions were immunoprecipitated as described in Material and Methods. The intensity of the bands was quantified and the maximal value was obtained at time cero and after 6 hours of chase for cytosolic and PM-associated IDE pools, respectively.

**Figure 2:**
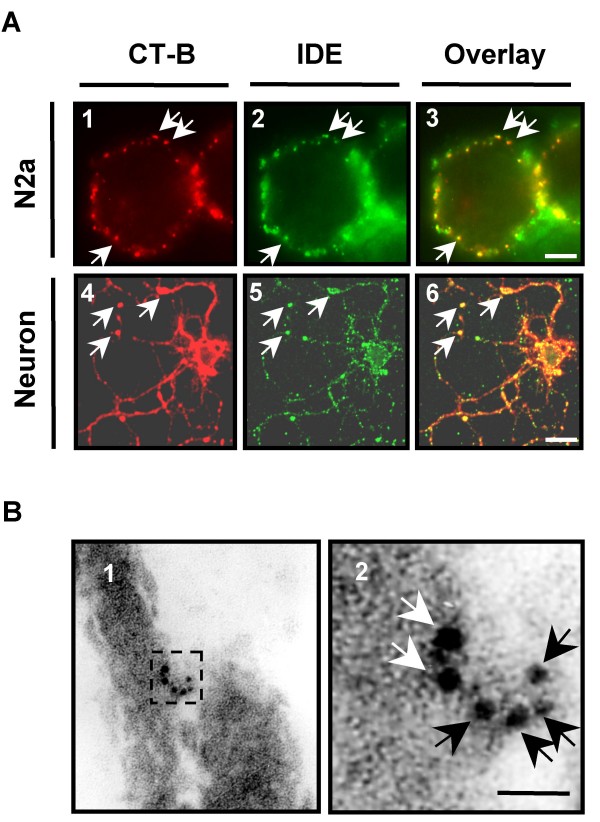
**Endogenous IDE is localized in DRMs of living cells**. **A**- Living N2aWT cells (panel 1–3) and primary hippocampal neurons (panel 4–6) were stained to label DRMs (panel 1 and 4) and IDE (panel 2 and 5). Merged images showed partial co-localization (arrows) on the PM (panel 3 and 6). Scale bar, 5 μm (panel 1–3) and 20 μm (panel 4–6).**B**- Immunogold electron microscopy on cryosections of N2aSW cells showed clusters of gold particles at the PM (panel 1). A higher magnification of the section framed in panel 1 (panel 2) clearly indicated co-localization of gold particles of different size corresponding to flotillin-1 (black arrows, 6 nm size) and IDE (white arrows, 15 nm size). Scale bar, 50 nm.

Immunocytochemistry without fixation and permeabilization using specific and well-characterized anti-IDE monoclonal antibodies (1C1 and 3A2) [17] in living N2a cells wild type (N2aWT) or stably expressing EGFP (N2aEGFP) showed a strong punctuated fluorescence restricted to the cell surface consistent with a PM-associated protein (Fig. 1A) as previously reported using cell surface biotinylation [26]. No reactivity was detected when purified IgG from normal mouse serum was used (not shown). Moreover, we confirmed this observation by immunoelectron microscopy on anti-IDE-stained cryosections of N2aSW cells (Fig. 1B1). A higher magnification of a portion (framed) of Fig. 1B1 showed a cluster of colloidal gold particles of homogenous size (arrows) corresponding to IDE (Fig. 1B2). These results are consistent with the notion that IDE is located facing the outer side of the PM. Pulse-chase experiments of metabollicaly labeled IDE showed that cytosolic IDE peaked at time zero of chase and decreased in 96 hours while membrane-associated IDE peaked after 6 hours of chase and decayed markedly in 24 hours (Fig. 1C). A marked difference was observed in the decay-rate with a half-life of 33 hours and 4 hours for cytosolic and membrane-associated IDE, respectively. Together our results indicate that endogenous membrane-IDE follows a definite targeting and clearance pathway that rules-out a contamination with cytosolic IDE in our membrane preparations. To further characterize PM-associated IDE we co-immunostained PM-IDE and DRMs in living N2aWT cells (Fig. 2A panels 1–3) and in hippocampal primary neurons (Fig. 2A panels 4–6). The pattern of reactivity showed individual red (CT-B) and green (IDE) spots distributed in the cell surface compatible with "patches" in the PM. We observed that IDE partially co-localized with CT-B-labeled DRMs (arrows) in both cell types. We determined that 14.7 ± 0.7% of PM-associated IDE co-localized with DRMs containing GM1 in N2aWT cells. To confirm that IDE resides in DRMs, we performed immunogold electron microscopy (IEM) on N2a cells using specific antibodies anti-IDE and anti-flotillin-1, a widely used marker of DRMs. A representative image is shown in Fig. 2B1. Higher magnification of the framed portion selected in Fig. 2B1 (Fig. 2B2) clearly indicated the presence of colloidal gold particles of different sizes corresponding to IDE (15 nm, white arrows) and flotillin (6 nm, black arrows). The co-localization of both antigens further indicates the presence of IDE in DRMs and it is consistent in with the "patch" staining observed by immunofluoresence.

### Endogenous IDE fractionates with DRMs isolated from human brain and N2a cells

**Figure 3:**
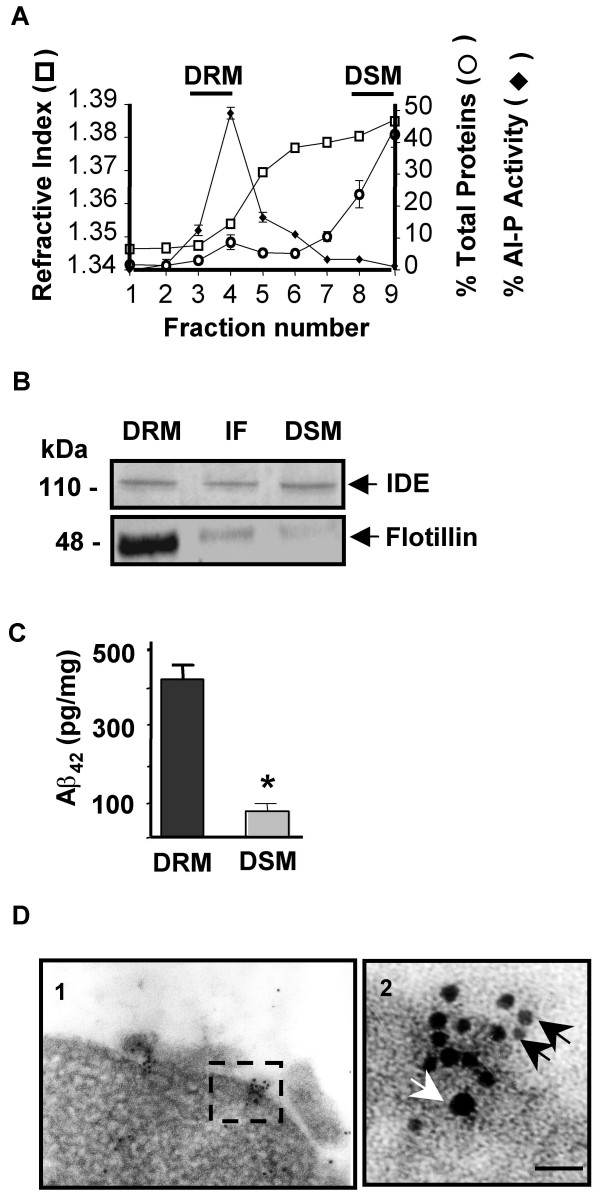
**Endogenous IDE co-localizes with flotillin-1 and Aβ on the plasma membrane**. **A**- Brain rat membranes were processed as described in Materials and Methods and fractions analyzed by refractive index (□), distribution of total protein (○) and GPI-anchored alkaline phosphatase activity (◆). DRMs, fraction 3–4; DSMs, fractions 8–9. Fraction 1, top of the gradient; fraction 9, bottom of the gradient.**B**- Representative western blotting of the same amount of total protein from DRMs and DSMs isolated from cortical tissue of a FAD brain showed co-localization of IDE and flotilin-1 in DRMs. IF, intermediate fraction. **C**- Significant increased amount of Aβ 42 was detected in DRM compared to DSM by ELISA. Bars represent means ± S.E.M of 2 independent experiments. *p < 0.001. **D**- Immunogold electron microscopy on cryosections of N2aSW cells showed clusters of gold particles at the plasma membrane (panel 1). A higher magnification of the section framed in panel 1 (panel 2) clearly indicated co-localization of gold particles of different size corresponding to Aβ (white arrow, 15 nm) and IDE (black arrows, 6 nm). Scale bar, 50 nm.

We next used a biochemical approach to characterize the association of IDE with DRMs. DRMs are sub-cellular compartments that although do not correspond to lipid rafts as they exist in vivo, their biochemical separation are the only approach for assessing protein interactions with lipid rafts [27,28]. N2a cell membranes were isolated, treated with detergent at 4°C and subjected to sucrose gradient centrifugation. All fractions from the sucrose density gradient were analyzed by total protein distribution, refractive index and GPI-alkaline phosphatase activity (Fig. 3A). In agreement with a previous report [25], most of the alkaline phosphatase activity was located at the 5–30% sucrose interface, representing the DRMs (fraction 3 and 4) while detergent-soluble membranes (DSMs) were limited to fractions 8–9. Interestingly, as shown in Fig. 3B IDE immunoreactivity was detected in DRM fraction together with flotillin-1 which is over-represented in DRMs as compared to DSM indicating that the biochemical fractionation of brain DRMs was properly performed. To further investigate the co-residence of IDE with Aβ in DRMs we isolated DRMs and DSMs from the cerebral cortex of a familial AD case (carrying the PS1 mutation M146L), known to present extensive Aβ accumulation [19]. The presence of Aβ42 in DRM and DSM was corroborated by sandwich ELISA assay (Fig. 3C). Substantial peptide concentration was detected in DRM as compared to DSM in agreement with previous reports [29]. Taking into account the technical impossibility to perform a triple-immuno gold electron microscopy to detect IDE, Aβ and flotillin we performed IEM on N2aSW cells to localize IDE and Aβ (Fig. 3D1). A higher magnification of the portion framed in Fig. 3D1 (Fig. 3D2) showed clusters of colloidal gold particles of different sizes corresponding to IDE (black arrows, 6 nm) and Aβ (white arrows, 15 nm) detected on the PM. The experimental evidences that Aβ levels are significantly increased in DRM as compared to DSM and the highly specificity of the immunogold strongly suggest that DRMs may be one of the sub-cellular compartments where IDE and Aβ interact.

### IDE isolated from DRMs of rat brain is proteolitically active in vitro

**Figure 4:**
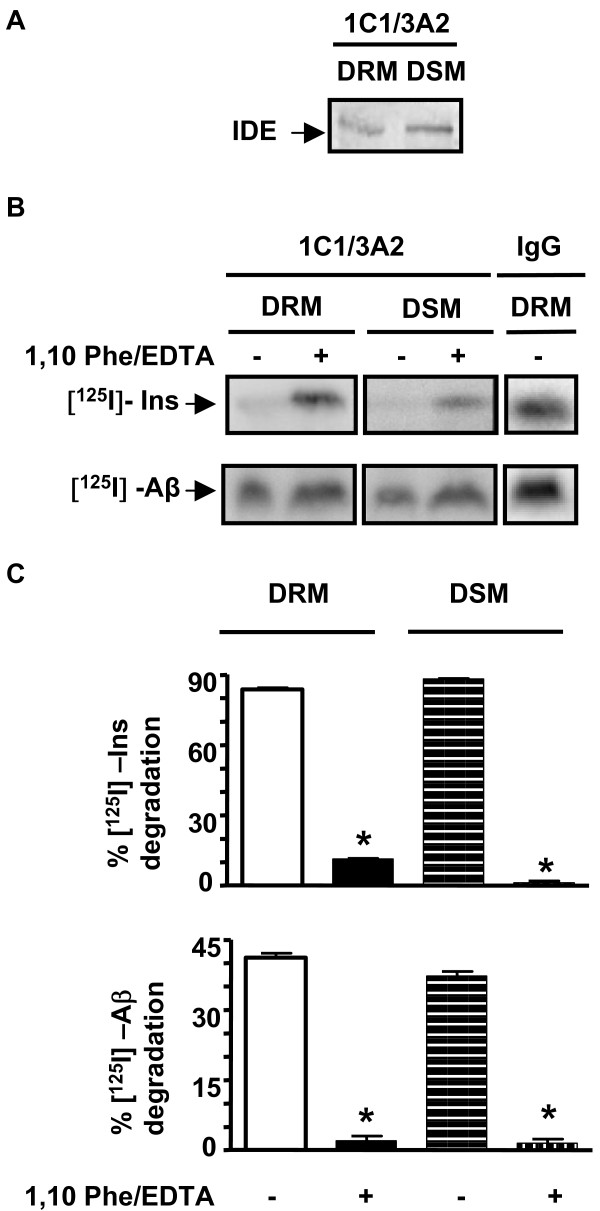
**DRM- and DSM-associated IDE pools are proteolytically active in vitro**. **A**- Western blotting with BC2 anti-IDE polyclonal antibody of immunoprecipated IDE from isolated DRM and DSM fractions of rat brain using 1C1/3A2 anti-IDE monoclonal antibodies. **B**- Representative phosphorimage scan showed degradation of [^125^I]-insulin and [^125^I]-Aβ after incubation with anti-IDE immunoprecipitates from DRM and DSM in the presence of a protease inhibitor cocktail (as defined in Antibodies and Chemicals) with or without metalloprotease inhibitors (EDTA/1,10 phenantroline). IgG, unrelated immunoglobulin used as a negative control for the immunoprecipitation. The intensity of the band in the presence of unrelated IgG and after incubation in degradation buffer was referred as intact substrate (0% degradation). **C**- Bars represent the semi-quantitative analysis of the percentage of [^125^I]-insulin and [^125^I]-Aβ degradation by IDE from DRMs and DSMs in the presence and absence of 1,10-Phe/EDTA (n = 3; *p < 0.001).

To determine if DRM-associated IDE was proteolytically active, we examined degradation of insulin (the most specific substrate for IDE) and Aβ (a relevant substrate for AD pathology) by an immunoprecipitation-based assay. DRM and DSM fractions were isolated from rat brain cortex, and endogenous IDE was immunoprecipitated using 1C1 and 3A2 monoclonal antibodies. The presence of IDE in immunoprecipitates was confirmed by western blotting using BC2 anti-IDE antibody (Fig. 4A). Immunoprecipitates were then incubated with [^125^I]-insulin or [^125^I]-Aβ40, and degradation analyzed by SDS-PAGE followed by phosphorimaging. The results showed substantial degradation of substrates when reactions were performed in the absence of 1,10 phenanthroline/EDTA (Fig. 4B). Conversely, addition of inhibitor strongly blocked degradation of insulin and Aβ, indicating substrate specificity of IDE in the reactions. The partial resistance of Aβ to degradation was likely due to the biophysical state of radiolabeled peptide and the reported resistance of aggregated Aβ to IDE proteolysis. Semi-quantitative analysis of the degradation assays indicated that insulin degradation (Fig. 4C, upper panel) in the absence of 1,10 phenantroline/EDTA was 83.8 ± 0.6% and 88.2 ± 0.3% in DRM and DSM, respectively, while a significant degradation impairment was detected in both fractions after IDE inhibition (11.3 ± 0.3% and 1.2 ± 0.6% in DRM and DSM, respectively; n = 3; p < 0.001). In addition, f [^125^I]-Aβ degradation (Fig. 4C, lower panel) in the absence of 1,10 phenantroline/EDTA was 41.2 ± 0.9% and 37.3 ± 0.9% in DRM and DSM, respectively and values were significantly decreased after the addition of metalloproteases inhibitors (2.0 ± 1.0% and 1.5 ± 0.9% in DRM and DSM, respectively, n = 3, p < 0.001). Our results clearly indicate that DRM and DSM -associated IDE are proteolitically active in vitro.

### Manipulation of membrane lipid composition disrupts IDE-DRM association and affects Aβ degradation in intact cells

**Figure 5:**
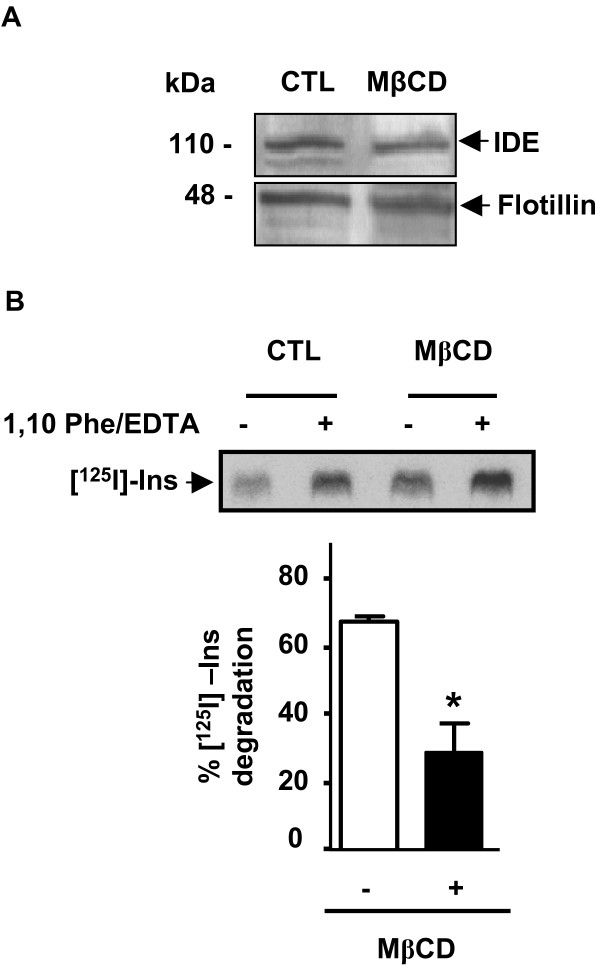
**PM-IDE activity in vitro is impaired after manipulation of membrane lipid composition**. **A**- Representative western blotting of IDE and flotillin-1 showed no differences in the protein levels in membranes isolated from control (CTL) and Mβ CD-treated cells.**B**- Representative PhosphorImage scan showed degradation of [^125^I]-insulin after incubation with membranes isolated from cells treated or not with Mβ CD in the presence of a protease inhibitor cocktail (as described in Antibodies and Chemicals) with or without metalloprotease inhibitors (1,10 phenantroline/EDTA). Bars showed the semi-quantitative analysis of the percentage of [^125^I]-insulin degradation by endogenous IDE from control and MβCD-treated membranes (n = 3; *p < 0.05).

**Figure 6:**
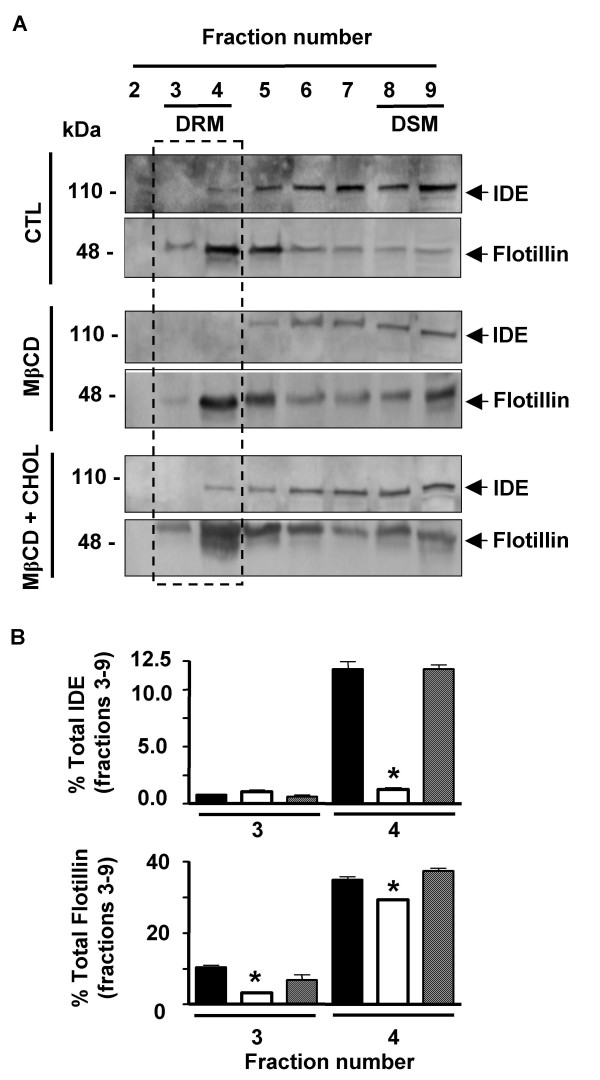
**Association of endogenous IDE with DRMs is sensitive to membrane-lipid composition**. **A**-Sucrose gradient fractions from N2aWT cells control (CTL), treated with MβCD alone (MβCD) or replenished with soluble Chol (MβCD + Chol) analysed by western blotting with anti-IDE and anti-flotillin-1 antibodies, respectively. Framed region, DRMs (fractions 3 and 4). **B**- Bars showed the percentage of total endogenous IDE and flotillin-1 in sucrose gradient fractions 3 and 4 (DRMs) from CTL (filled bars), MβCD (open bars) and MβCD + Chol (dashed bars). IDE and flotillin-1 were dramatically affected by membrane-lipid manipulation (n = 3; *p < 0.05 as compared to CTL values in each fraction). Fraction 2: top of the gradient. Fraction 9: bottom of the gradient.

Taking into account that des-localization of a given protein within a DRM as a consequence of lipid manipulations strongly suggests lipid raft association in vivo, we determined the effect of Chol, sphingolipids (SL) and phosphatitylcholine (PC) removalupon the association of IDE with DRMs. N2aWT cells were treated with MβCD under experimental conditions reported to be "mild", without associated cytotoxicity [30]. Membrane fractions from control and MβCD-treated cells showed similar amounts of Chol determined by an analytical procedure (1.26 μg/mg vs. 1.46 μg/mg) and similar amounts of IDE and flotillin (Fig. 5A) with a sharp reduction in insulin degradation from MβCD-treated cells which was statistically significant as compared to control cells (28.6 ± 8.5% vs. 66.9 ± 1.5%; n = 3; p < 0.05) (Fig. 5B). To find a biochemical explanation for these results we determined the membrane lipid composition from control and MβCD-treated cells by thin-liquid chromatography (TLC). Our data showed a decrement in the percentage of PC and SL in membranes from MβCD-treated as compared to control cells (30% and 2.39% vs. 44% and 4.25%). In addition, we showed lipid-dependent changes in IDE- and flotillin-DRM association (Fig. 6A, framed region). In this regard, DRMs (fraction 3 and 4) from untreated cells (Fig. 6B upper panel, black bar) retain 12.6 ± 0.6% of total membrane-associated IDE and 45.2 ± 1.1% of total membrane-associated flotillin (Fig. 6B lower panel, black bar) while no reactivity for IDE was detected in DRMs of MβCD-treated cells (Fig. 6B upper panel, white bar) and only a 32.5 ± 0.6% (n = 3; p < 0.05) of flotillin reactivity was observed (Fig. 6B, lower panel, white bar). Lipid manipulations induced a rise of IDE immunoreactivity in "intermediate fractions" (fractions 6–8) from 47.4 ± 2.3% to 66.2 ± 1.7%.

**Figure 7:**
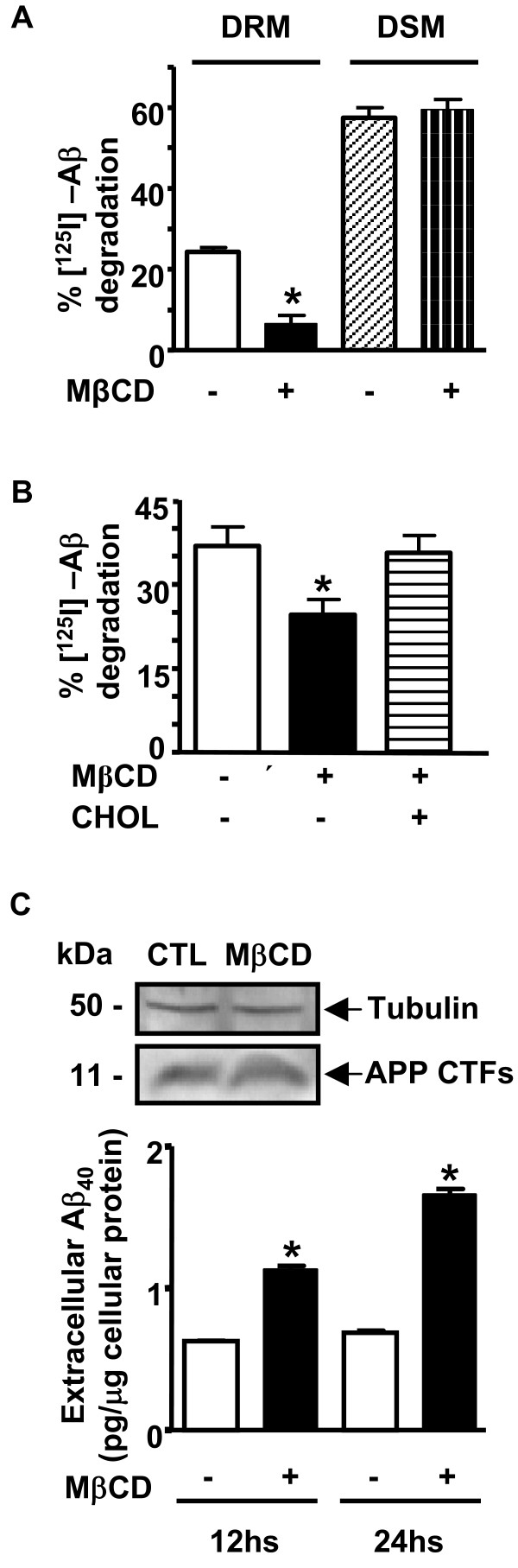
**Effect of disorganized DRM on Aβ degradation mediated by IDE**. **A**- Bars showed percentage of degraded [^125^I]-Aβ by IDE immunoprecipitated from DRM and DSM fractions from membranes treated or not with MβCD (n = 2; *p < 0.05). **B**- Bars showed the semi-quantitative analysis of the percentage of degraded extracellular [^125^I]-Aβ in the conditioned media of control, MβCD treated and Chol replenished N2aWT cells (n = 6; *p < 0.05). **C**- Upper panel, representative western blotting of C-terminal APP and tubulin levels from N2aSW cellular homogenates showed no differences between control (CTL) and MβCD-treated cells. Lower panel, bars represented Aβ 40 levels in the supernatants of CTL and MβCD-treated cells after 12 and 24 hours of incubation (n = 2; *p < 0.05).

Re-localization of IDE and flotillin to DRM in a percentage similar to control cells was obtained when water-soluble Chol was added to MβCD-treated cells (Fig. 6B upper and lower panels, dashed bar). Moreover, we immunoprecipitated IDE from DRMs and DSMs of control and MβCD-treated cells and incubated the immunoprecipitates with exogenous [^125^I]-Aβ. As it is shown in Fig. 7A a significant decrement of IDE activity was observed in DRMs from MβCD-treated cells as compared to control (6.55 ± 2.05% vs. 24.3 ± 1.1%; n = 2; p < 0.05) while no significant differences were detected in DSMs fractions from treated and control cells, respectively (57.5 ± 2.5% vs. 59.5 ± 2.7%; n = 2; p > 0.05). To assess if IDE mis-location from DRMs affects Aβ clearance, N2aWT intact cells were treated or not with MβCD in the presence of protease inhibitors as described above and exposed to nM concentrations of exogenous [^125^I]-Aβ. A significant reduction in [^125^I]-Aβ degradation was observed in MβCD-treated cells as compared to control (24.1 ± 2.0% vs. 36.9 ± 3.3%; n = 6; p < 0.05) (Fig. 7B). The addition of water soluble Chol to MβCD-treated cells restored the degradation profile to values similar to control cells (35.6 ± 3.1%, n = 6) indicating that DRMs-associated IDE is involved in the degradation of exogenous Aβ in living cells.

To determine the role of DRM-associated IDE on the steady-state levels of endogenous Aβ, we incubated N2aSW cultures in the presence or absence of MβCD as described above and the supernatant collected after 12 and 24 hours, respectively, for extracellular Aβ40 quantification. The experiments were performed in the presence of phosphoramidon, thiorphan and leupeptin to avoid NEP, ECE or plasmin activity. As depicted in Fig. 7C the extracellular levels of Aβ40 progressively accumulated after lipid efflux from the plasma membrane reaching values of 1.13 ± 0.02 and 1.66 ± 0.03 pg/μg of cellular protein as compared to control cells (0.62 ± 0.005 and 0.69 ± 0.01 pg/μg of cellular protein) after 12 h and 24 hours, respectively, (p < 0.05; n = 2). No significant change was observed in the levels of APP C-terminal fragments (Fig. 7C, inset) ruling out the unlikely possibility of an increased Aβ production during the incubation. Together with the in vitro results, these evidences suggested that the steady-state level of Aβ was significantly modified when IDE activity was displaced from DRMs.

### Brain cholesterol levels modulate IDE-DRM association in vivo

**Figure 8:**
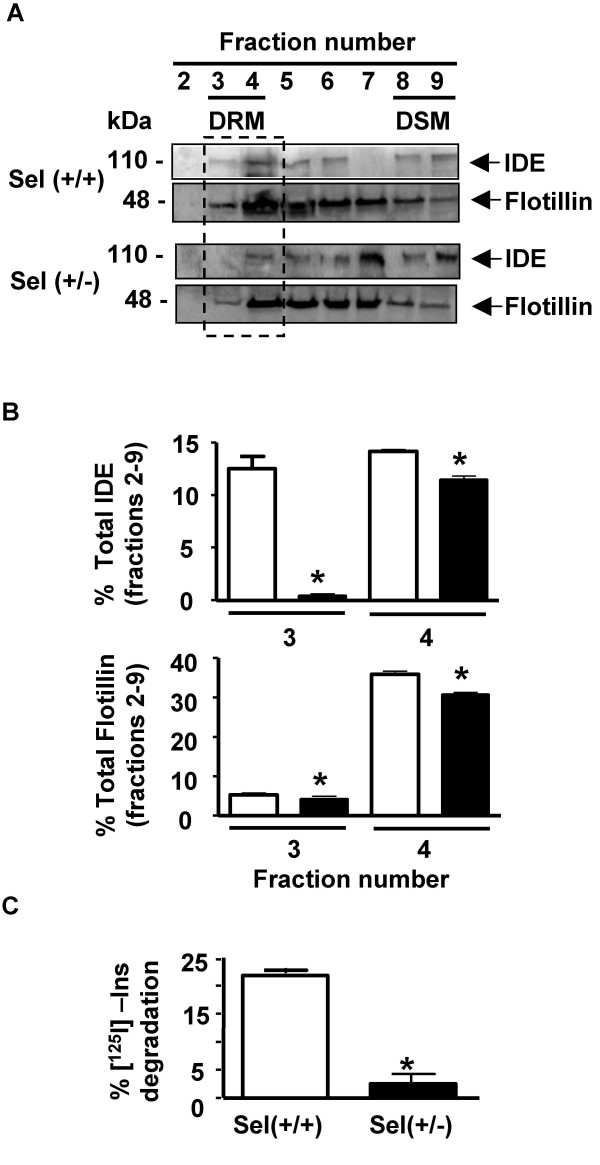
**Impact of brain cholesterol levels on endogenous IDE localization and activity**. **A**-Sucrose gradient fractions from sel-1 (+/+) and (+/-) mouse brains analyzed by western blotting with anti-IDE and anti-flotillin, respectively. Framed region, DRMs (fractions 3 and 4). Fraction 2: top of the gradient. Fraction 9: bottom of the gradient. **B**- Upper panel, bars showed the semi-quantitative analysis of the percentage of total endogenous IDE and flotillin-1 immunoreactivity in sucrose gradient fractions 3 and 4 (DRMs) from control (open bars) and partially knocked-out mice (filled bars). IDE and flotillin-1 present in DRM fraction were significant affected in sel-1 (+/-) mice brain compared to (+/+) mice (n = 3; *p < 0.05). **C**- Bars showed the semi-quantitative analysis of the percentage of [^125^I]-insulin degradation by endogenous DRMs-bound IDE from sel (+/+) and sel (+/-) brain membranes in the presence of a protease inhibitor cocktail as described in Antibodies and Chemicals (n = 2; *p < 0.05).

To determine the impact of brain Chol levels on IDE localization in vivo, mice with a targeted deletion of one seladin-1 (sel-1) allele (+/-) were used. Membranes from sel-1 (+/-) mouse brains showed reduced levels of Chol as compared to wild type sel-1 (+/+) mice (2.57 μg/mg vs. 5.55 μg/mg) and disorganized DRMs [31]. The flotation profiles of IDE and flotillin from sel-1 (+/+) mouse brains (Fig. 8A, upper panel) showed no significant differences as compared to rat or human brain as described above. In agreement with a DRM-specific shortage, reduction of brain sel-1 levels resulted in the displacement of IDE and flotillin (Fig. 8A, lower panel) from DRM. Thus, while in sel-1 (+/+) mice 27.7 ± 1.36% of the total IDE and 41.2 ± 0.76% of total flotillin resides in fractions 3 and 4, respectively, only 11.9 ± 0.4% and 34.8 ± 0.85% (n = 3; p < 0.05) of IDE and flotillin, respectively, was observed in sel-1 (+/-) brains (Fig. 8B). Decreased Chol levels induced a rise of IDE immunoreactivity in "intermediate fractions" (fractions 5–7) of sel (+/-) as compared to sel (+/+) mice brains (62.0 ± 1.3% vs. 28.3 ± 1.5%, respectively). As expected, a drop in [^125^I]-insulin degradation by IDE in sel-1 (+/-) was observed as compared to sel-1 (+/+) brains (2.6 ± 1.7% vs. 21.9 ± 0.9%; n = 2; p <0.05) (Fig. 8C).

## Discussion

Together our results of live immunofluorescence on unpermeabilized cells, immunoelectron-microscopy and gradient fractionation concurred to the demonstration that IDE is, in addition to its other localizations, a DRM-associated metallopeptidase. Noteworthy, the analysis of IDE primary structure shows no evidence for membrane-binding sequences, no post-translational modifications are known and our metabolic labeling studies (not shown) with [^3^H]-palmitate and [^3^H]-ethanolamine did not support palmitoylation or GPI anchor (Morelli L, unpublished observations). However, the existence of DRM-associated proteins lacking these motifs has been demonstrated. In these cases, inclusion into DRMs was postulated to be mediated by mechanisms involving protein-protein interactions [32], direct association through post-translational modifications [33] or changes in the conformational state of the cytosolic protein [34]. IDE may be anchored directly to DRMs by a yet uncharacterized post-translational modification or indirectly through a strong interaction with a protein residing in these structures. The specificity of our monoclonal antibodies together with the degradation assays in intact cells indicated that at least the IDE N-terminal domain including the catalytic site had a fully functional extracellular location. Taking into account the requirement of the C-terminal domain for substrate binding [35] it is likely that the entire IDE molecule is located on the cell surface. This notion is in accordance with previous work showing the cell surface biotinylation of IDE [26] and the more recent demonstration that IDE may act as a cellular receptor for Varicella Zoster virus [36].

Results from the pulse chase experiments are in accordance with the existence of at least two pools of cellular IDE. The cytosolic one with a longer half-life as previously reported [37] and the membrane-IDE with faster turn-over, in agreement with reports described for DRM resident proteins [38,39]. Significant differences between the t1/2 of both pools of IDE are predictable and reasonable taking into account that each pool may undergo different degradative pathways.

Our finding that a pool of active IDE is associated with DRMs is highly relevant to Aβ and insulin metabolism. DRMs appear to be central to the process of Aβ aggregation in the course of the disease, as suggested by AD animal models [29]. The co-fractionation of IDE activity and Aβ in DRMs in addition to the evidence that Aβ degradation is impaired by DRMs disruption raises the possibility that a substantial part of Aβ proteolytic removal may take place at DRMs. This hypothesis is further supported by the fact that two other proteases involved in Aβ degradation -NEP and plasmin- are also present in DRM [22,29]. Here we show that MβCD treatment resulted in a decrement of the PC and SL levels associated with a significant displacement of IDE from DRMs with a reduction on Aβ clearance. PC is the main cell glycerophospholipid, and it is the main lipid component of lipid membrane domains from cerebelar neurons [40]. Thus the reduction in the content of PC can be considered a good indication of a general reduction of the surface area occupied by the lipid domain. Our experimental evidences propose that lipid content in DRMs may modulate not only Aβ generation [41,42] but also its degradation. In this regard, it was previously reported that Chol depletion by combination of lovastatin (which decreases de novo synthesis of Chol) and MβCD (which promotes lipid efflux from the plasma membrane), inhibits Aβ secretion in hippocampal neurons, probably due to changes in the intracellular transport of APP, enhanced APP α-cleavage and decreased APP β-cleavage, respectively [41,43,44]. Moreover, the Aβ-lowering effect of Chol manipulations was not observed in COS cells stably transfected with human APP695 [41]. In addition, it was reported that Chol depletion of membranes from CHO cell lines stably co-expressing wild type human APP and presenilins, did not affect the γ-secretase activity present in DRMs [45]. In this context, our results suggest that the sole efflux of Chol, PC and SL from the plasma membrane, without the effect of inhibiting Chol synthesis, probably does not affect APP processing and the consequent Aβ generation but instead may negatively impact on Aβ clearance mediated by DRMs-associated Aβ proteases. The evidences that Aβ clearance is impaired when IDE is shifted from DRMs, even if DSM-associated IDE is proteolytically active in vitro, suggest an important role of membrane lipid composition in the activity of enzymes associated with membranes in living cells. It is possible that membrane-associated IDE conformational requirement and its interaction with other proteins for substrate degradation are only optimal in the context of organized DRMs. This may explain why, in our experiments in vitro using membranes isolated from MβCD-treated cells or from mouse brain of sel-1 (+/-) mice, a shift of IDE from DRMs (82% in MβCD-treated cells and 55.5% in sel-1 (+/-) mouse brain) induced a substantial decrement on IDE proteolytic activity (73% and 88% in MβCD-treated cells and sel-1 (+/-) mouse brain, respectively).

Alternatively, DRMs but not DSMs, may act as platforms where Aβ accumulates, due to its hydrophobic properties, reaching local concentration close to its Km for IDE. Our experiments with intact cells where the extracellular concentration of Aβ was approximately 0.2 nM (far below the known Km of IDE for Aβ~1–2 μM) showed that the degradation was inefficient in intact cells pre-treated with MβCD. It was previously reported that when the loss of lipids is relative limited, as the case of a "mild" MβCD treatment, the membrane lipids change their membrane organization, reducing the total surface area of membrane lipid domains. This can be achieved by reducing the total number of domains or their sizes [46]. The accumulation of endogenous Aβ after lipid manipulations further supports this possibility. Although a broad protease inhibition was used (including NEP, ECE and plasmin), we cannot rule out a contributory effect of matrix metallo peptidases [47]. Alternatively, reduced binding and uptake of Aβ due to lipid manipulation of the PM needs to be further examined as a possible contributory mechanism of Aβ accumulation.

Taking into account that it was previously demonstrated that only moderate levels of desmosterol accumulate in brains of sel-1 (+/-) mice and that the exogenous addition of desmosterol to neuroblastoma cells has no effect in the flotation profile of DRMs enriched proteins [31] we assume that the differences observed in IDE proteolytic activity between sel-1 (+/+) and sel-1 (+/-) brains were due to lower Chol in DRMs rather than to an inhibitory effect of desmosterol. These results are in agreement with the evidence that in sel-1 (+/-) mice, steady-state levels of brain Aβ are significantly elevated, and human Aβ accumulates in sel-1 (+/-) mice bred to transgenic mice overexpressing APP harboring the Swedish mutation [31].

Our data of the relevance of DRMs integrity in IDE mediated proteolysis are in accordance with the behavior of the insulin receptor (IR) which, after Chol depletion, is able to interact with insulin but unable to propagate the insulin signaling cascade due to its mis-location from the DRMs [48]. In this scenario, our results lead us to suggest that structural integrity of DRMs may be required to tightly retain IR and IDE for at least 2 functions: 1- further downstream insulin signaling and 2- insulin proteolysis once in the endocytic vesicle which contains receptor, ligand and enzyme.

The relationship among DRMs integrity, lipid content, sel-1 expression and AD pathology has been extensively reported [22,31,49-51] and alterations of DRM composition in the course of AD have been described [52]. Moreover, abnormal DRM lipid composition has been suggested as a molecular mechanism involved in insulin resistance, thought to be the primary defect in the pathophysiology of type 2 diabetes [53]. In this regard, it is noteworthy that disturbances in insulin metabolism, especially insulin resistance, may play a role in pathogenic processes that promote the development of sporadic AD. Individuals suffering from AD had been shown to have lower cerebrospinal fluid and higher plasma insulin concentration [54]. Since insulin has been shown to affect Aβ and IDE levels and tau hyperphosphorylation in the brain, this issue has recently emerged as a novel field of sporadic AD ethiopathogenesis and therapy research [55].

## Conclusion

It has been suggested that protease-mediated Aβ clearance becomes inefficient with aging [56] and the impact of a reduction in DRM-associated IDE may be particularly significant. Minor changes in Aβ levels may dramatically affect its oligomerization rate with the consequent escape of Aβ from the physiologic pathway of proteolytic removal. The concept that mis-location of IDE and other Aβ degrading proteases away from DRMs may impair the physiological turn-over of Aβ in vivo deserves further investigation in light of therapeutic strategies based on enhancing Aβ proteolysis in which DRM protease-targeting may need to be taken into account.

## Competing interests

The authors declare that they have no competing interests.

## Authors' contributions

All authors read and approved the final manuscript. AB acquired, analyzed and interpreted data and helped to draft the manuscript and figures. MCL performed the primary cultures of hippocampal neurons and participated in the set up of the immunogoldelectron microscopy. EIS carried out the determination of cytosolic and plasma membrane-associated IDE turn-over. XZ helped to draft the manuscript. HX contributed towards experimental design, partially supported AB stage in his laboratory, supplied cells and regents critically for the study and helped to draft the manuscript. MDL. provided sel-1 (+/-) and sel-1 (+/+) brain tissues and helped to draft the manuscript. EMC collaborated in the study design, in the interpretation of the data and in the draft of the manuscript. LM oversaw the experimental design, data analysis, data interpretation, and drafting/editing the manuscript.
